# Supplementary material for: Preterm Infants Harbour a Rapidly Changing Mycobiota That Includes Candida Pathobionts
Source: J Fungi (Basel). 2020 Nov 9;6(4):273. doi: 10.3390/jof6040273 (PMC7712117; doi:10.3390/jof6040273)
Supplement: Supplementary file 1 [file jof-06-00273-s001.zip › James et al_Supplementary/James et al_Table S6.pdf]

**Table S6.** Persistent fungal species, re-occurring in 2 or more infant longitudinal samples.

| Species* | Category** | Grows at 37C | Infant | | | | | | | | | | |
|---|---|---|---|---|---|---|---|---|---|---|---|---|---|
| | | | F1 | F2 | F3 | F4 | F5 | M1 | M2 | M3 | M4 | M5 | M6 |
| *Aspergillus ruber* | Environmental | + | | | | | | ■ | | | | | |
| *Aspergillus subservicolor* | Environmental | - | ■ | | | | | | | | | | ■ |
| *Baeospora myosura* | Environmental | - | ■ | ■ | ■ | ■ | ■ | ■ | ■ | ■ | ■ | ■ | ■ |
| *Candida albicans* | Human-associated | + | ■ | ■ | ■ | | | ■ | ■ | ■ | | | ■ |
| *Candida parapsilosis* | Human-associated | + | ■ | ■ | ■ | ■ | ■ | ■ | ■ | ■ | ■ | ■ | ■ |
| *Candida tropicalis* | Human-associated | + | ■ | | | | | ■ | | | | | |
| *Davidiella tassiana* | Plant pathogen | - | ■ | ■ | ■ | | ■ | ■ | ■ | ■ | ■ | ■ | ■ |
| *Debaryomyces hansenii* | Food-related | v | ■ | | ■ | | | ■ | | ■ | | | ■ |
| *Filobasidium magnum* | Environmental | - | | | | | | | | ■ | | | ■ |
| *Hypholoma fasciculare* | Environmental | - | ■ | | | | | | | | | | |
| *Malassezia restricta* | Human-associated | v | | ■ | ■ | | | ■ | | | | ■ | |
| *Meyerozyma guilliermondii* | Human-associated | + | ■ | | | | | | | | | | ■ |
| *Monilinia laxa* | Plant pathogen | - | ■ | | | | | | | | | | |
| *Naganishia diffluens* | Environmental/Human-associated | - | | | | | | | ■ | ■ | | | |
| *Penicillium astrolabium* | Environmental | - | ■ | | | | | | | | | | |
| *Penicillium glabrum* | Plant pathogen | - | | ■ | | | ■ | | | | | | |
| *Penicillium polonicum* | Plant pathogen | - | | | | ■ | | | | | | | |
| *Periconia cookei* | Plant pathogen | ? | ■ | | | | | | | | | | |
| *Rhodotorula mucilaginosa* | Environmental/Human-associated | - | | | | | | ■ | ■ | | | ■ | ■ |
| *Saccharomyces cerevisiae* | Food-related | v | ■ | | ■ | | ■ | | | ■ | | | ■ |
| *Talaromyces infraolivaceus* | Environmental | - | | | | | ■ | | | | | | |
| *Vishniacozyma carnescens* | Environmental | - | | | | | | ■ | | | | | |
| *Wickerhamomyces onychis* | Human-associated | + | | | | | ■ | | | | | | |

* Opportunistic human pathogens are shown in red.

■ Present in 2 or more longitudinal samples

** *N. diffluens* , skin commensal (occassional); *Rh. mucilaginosa* , gut commensal (putative).
