# Supplementary material for: Preterm Infants Harbour a Rapidly Changing Mycobiota That Includes Candida Pathobionts
Source: J Fungi (Basel). 2020 Nov 9;6(4):273. doi: 10.3390/jof6040273 (PMC7712117; doi:10.3390/jof6040273)
Supplement: Supplementary file 1 [file jof-06-00273-s001.zip › James et al_Supplementary/James et al_Table S7.pdf]

Table S7. Relative abundance values of *Debaryomyces hansenii* and *Saccharomyces cerevisiae* at 6-, 12- and 18-months.

Infant Sample:	*D. hansenii*	*S. cerevisiae*
F1_06	5.24	0.50
M1_06	0.00	0.00
F2_06	0.00	0.00
F3_06	0.00	0.00
M3_06	11.71	0.92
F4_06	0.00	0.00
F5_06	0.00	0.37
M5_06	0.00	0.00
M6_06	2.17	0.00
F1_12	99.37	0.00
M1_12	0.74	73.33
M2_12	0.00	0.00
F2_12	0.65	0.82
F3_12	40.16	15.72
M3_12	0.00	9.58
F4_12	0.00	0.00
M4_12	0.03	0.00
F5_12	0.00	64.91
M5_12	0.00	0.00
M6_12	0.12	51.53
F1_18	5.01	0.98
M1_18	1.39	0.00
M2_18	0.00	0.05
F3_18	0.02	95.85
M3_18	4.23	0.09
M4_18	0.00	0.01
F5_18	0.00	0.00
M5_18	0.00	0.00
M6_18	2.09	3.23

Mean relative abundance:
D. hansenii : 2.13%
S. cerevisiae : 0.2%

Mean relative abundance:
D. hansenii : 12.82%
S. cerevisiae : 19.63%

Mean relative abundance:
D. hansenii : 1.42%
S. cerevisiae : 11.13%
