# Supplementary material for: Preterm Infants Harbour a Rapidly Changing Mycobiota That Includes Candida Pathobionts
Source: J Fungi (Basel). 2020 Nov 9;6(4):273. doi: 10.3390/jof6040273 (PMC7712117; doi:10.3390/jof6040273)
Supplement: Supplementary file 1 [file jof-06-00273-s001.zip › James et al_Supplementary/James et al_Table S5.pdf]

Table S5. Most abundant fungal species.

Species*	Category	Mean abundance (%)	Prevalence (%)**
Baeospora myosura	Environmental	19.75	93.1
Candida parapsilosis (Y)	Human-associated	13.28	89.7
Saccharomyces cerevisiae (Y)	Food-related	10.96	51.7
Candida albicans (Y)	Human-associated	10.89	65.5
Debaryomyces hansenii (Y)	Food-related	5.96	48.3
Davidiellaa tassiana	Plant pathogen	5.50	79.3
Naganishia diffluens (Y)	Human-associated	2.82	27.6
Candida metapsilosis (Y)	Human-associated	2.42	13.8
Penicillium polonicum	Plant pathogen	2.15	24.1
Pleurotheciella rivularia	Environmental	1.99	10.3

* (Y), denotes a yeast species

** n = 29
