# Supplementary material for: Preterm Infants Harbour a Rapidly Changing Mycobiota That Includes Candida Pathobionts
Source: J Fungi (Basel). 2020 Nov 9;6(4):273. doi: 10.3390/jof6040273 (PMC7712117; doi:10.3390/jof6040273)
Supplement: Supplementary file 1 [file jof-06-00273-s001.zip › James et al_Supplementary/James et al_Table S2.pdf]

Table S2. Number of fungal taxa detected in each infant faecal sample.

Infant:	Sample:		
	6-month (X_06)	12-month (X_12)	18-month (X_18)
F1	23	5	31
F2	12	10	N/A
F3	7	13	20
F4	8	20	N/A
F5	7	14	28
M1	28	37	12
M2	N/A	18	10
M3	15	11	14
M4	N/A	5	12
M5	5	9	12
M6	16	28	27
**Mean**	**13.4**	**15.5**	**18.4**

N/A, Sample unavailable.

Note: Only taxa with relative abundance values of 0.01% or greater were accepted (i.e. detection threshold cut-off).

Overall mean (all samples): **15.8**
