# Supplementary figures and images for: Preterm Infants Harbour a Rapidly Changing Mycobiota That Includes Candida Pathobionts
Source: J Fungi (Basel). 2020 Nov 9;6(4):273. doi: 10.3390/jof6040273 (PMC7712117; doi:10.3390/jof6040273)

Alpha-diversity Index: Shannon

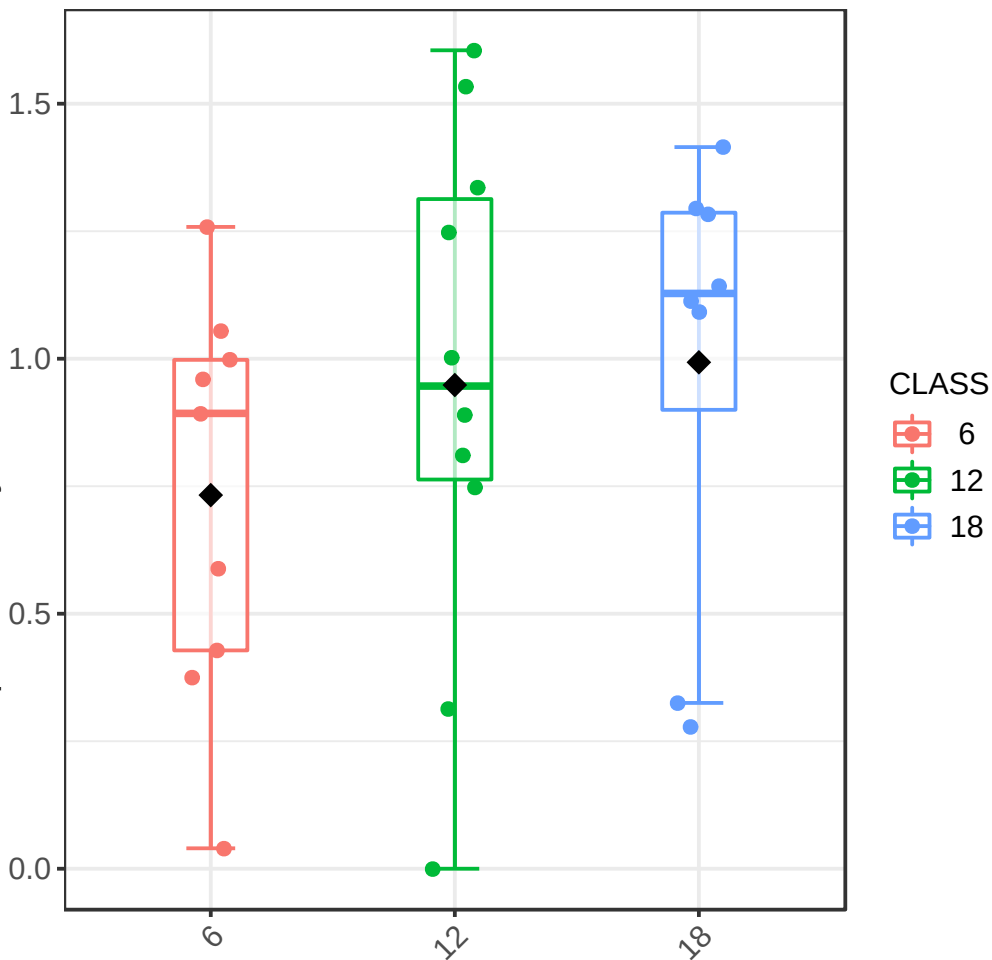

Supplement: Supplementary file 1 [file jof-06-00273-s001.zip › James et al_Supplementary/James et al_Fig.S3.pdf]

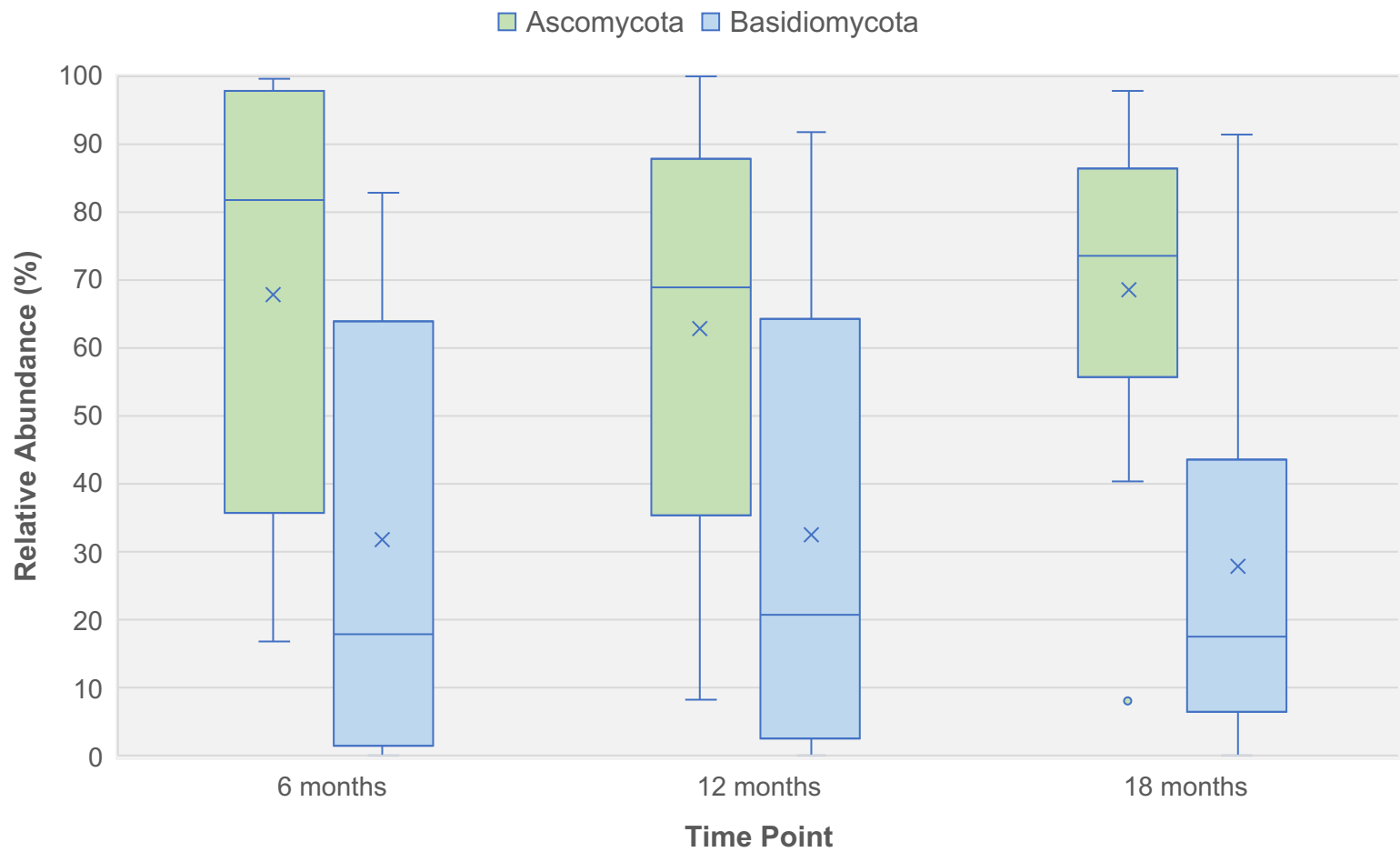

Supplement: Supplementary file 1 [file jof-06-00273-s001.zip › James et al_Supplementary/James et al_Fig.S2.pdf]

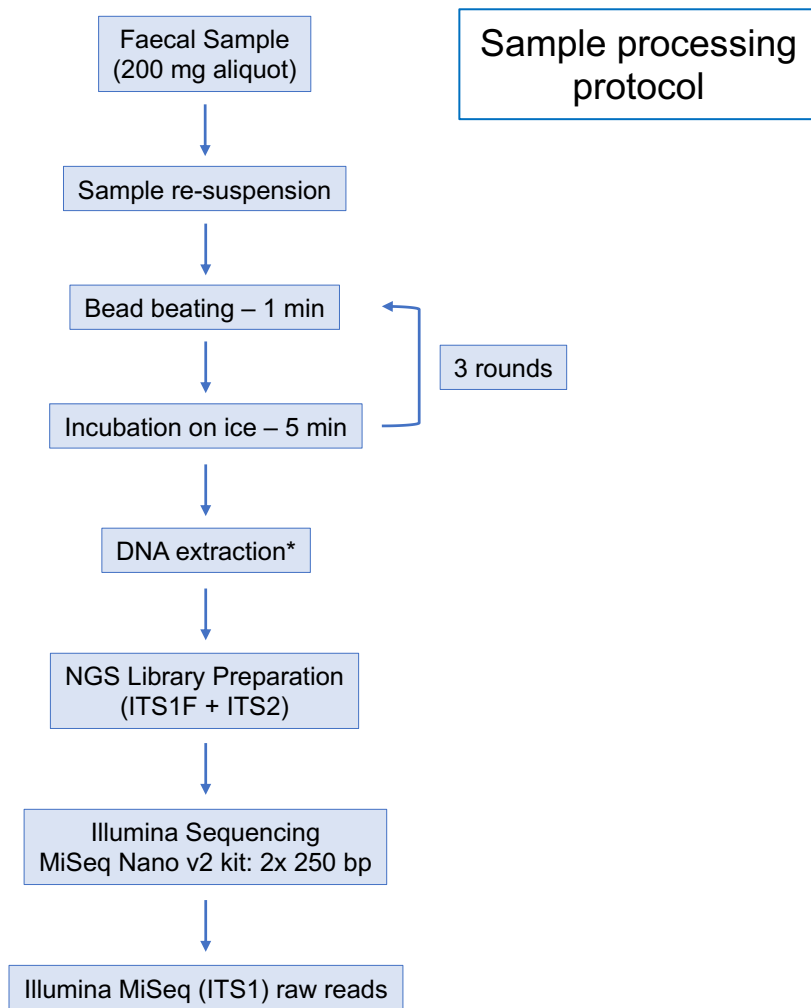

\*Using the MP FastDNA Spin kit for Soil

## Bioinformatics pipeline

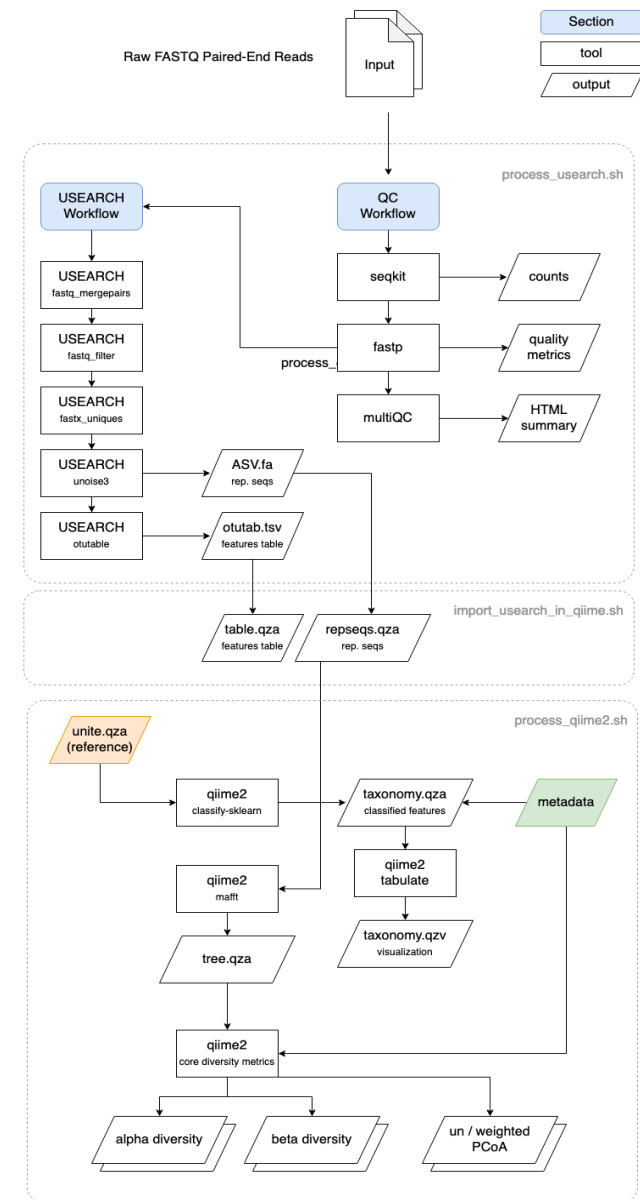

Supplement: Supplementary file 1 [file jof-06-00273-s001.zip › James et al_Supplementary/James et al_Fig.S1.pdf]
